# Notes on Preparations for the Sick

**Published:** 1907-12

**Authors:** 


### Notes on Preparations for the Sick.

**Kuhn's Suction Mask.**—Roborat Company, 8 Harp Lane, London, E.C.—For the congestive treatment of pulmonary tuberculosis and asthma, in accordance with Prof. Bier's system of artificial hyperæmia. The mask is made of celluloid, the mouth and nose portions being separate chambers. When applied to the face by means of the elastic band (which should be fastened round the neck under the ears), both nose and mouth are completely shut in. The air tube should be always well inflated, a tight constriction being placed around the small rubber tube after blowing up. A trial may then be made with the sliding valve in the nose portion completely closed, when respiration will be found to be so much impeded as to become impossible. As a general rule, breathing should take place only through the nose, though in case of impediment arising from cold or other cause, air may be made to enter the mouth by opening the sliding valve in the cross partition. Expiration occurs unhindered through the circular valve provided for the purpose in both portions of the apparatus, and, with practice, can be made to take place quite freely through the mouth even with the valve aperture somewhat diminished. The aim of the use of the mask is, by providing a resistance to the intake of air into the lungs, to bring about an increased negative pressure in the thorax and thereby suction of blood into the lungs. The greater the resistance, therefore, and the longer it is continued, the greater becomes the amount of blood flowing into the lungs. It should not, however, be so great or so long continued as to bring on unpleasant by-effcts, such as headache or other discomfort. It is quite possible that during the first few days of use there may be some singing in the ears, in which case it is recommended that the mouth be closed, the nose held, and the cheeks inflated, when it will be found that air drawn out of the middle ear while wearing the mask is replaced through the Eustachian tubes. Another simple remedy is to make a few purposeful swallowing movements. This symptom is not of frequent occurrence, and in any case disappears after a few days. Inspiration against resistance by means of this method should be an active agent in the treatment of anæmic or tuberculous subjects. An increase in the number of red blood corpuscles is brought about, and an increased hyperæmia should have a beneficial influence upon the upper parts of the lungs which are most prone to tuberculosis. Good results have been obtained by Prof. von Leyden at the Berlin Charité.

---

**Formidine: Methylen Disalicylic Acid Iodide.**—PARKE, DAVIS & Co., London.—Formidine is an internal and external antiseptic and a substitute for iodoform. It is a true chemical compound, a condensation product of iodine, formic aldehyde, and salicylic acid, having the formula $C_{15}H_{10}O_6I_2$. It may be called an iodised derivative of salicylic acid and formic aldehyde, exhibiting none of the physical characteristics nor responding to any of the chemical tests for its constituent bodies until decomposed. Formidine is a reddish-yellow powder, tasteless, and having a slight but not disagreeable odour suggestive of iodine; it contains about 47 per cent. of this element. It is perfectly stable in the dry state. When heated, iodine is liberated, the substance melts, then chars, and is finally consumed without leaving any ash. It is insoluble in water, dilute acids, alcohol, and most other ordinary solvents, and does not decompose with glycerin. When brought into contact with alkaline organic secretions it slowly dissolves and develops the characteristics of its constituents, iodine, salicylic acid, and formic aldehyde. It is supplied in five grain capsules for internal administration, and in this form it appears to be one of the best of intestinal antiseptics.

---

**Egmol.**—PARKE, DAVIS & CO., London.—This preparation contains 40 per cent. of finest olive oil emulsified with fresh eggs and flavoured with best French brandy. It is a nutritious and stimulating food, which may be prescribed with excellent effect in wasting diseases. For those patients who cannot tolerate cod liver oil, egmol forms an excellent substitute ; indeed, experiments carried out by Cunningham (vide *Journal of Physiology*, xxiii. 209) seem to show that owing to more perfect assimilation, olive oil may be ranked above cod liver oil as a flesh former and heat producer. Egmol is a perfect and stable emulsion, and in every way an excellent example of good pharmacy.

---

**Petroleum Emulsion.**—PARKE, DAVIS & CO., London.—In spite of its slight utility, there has arisen a demand for an emulsion of petroleum. Much of the liquid paraffin of commerce is undesirable for medicinal use, but in the P. D. & Co. petroleum emulsion a pure fluid hydrocarbon is used, which is wholly free from acid traces and from the sulphur compounds often present in imperfectly refined liquid paraffin, and with it are associated suitable amounts of the hypophosphites of calcium and sodium.

---

**Adrenalin and Chloretone Ointment.**—PARKE, DAVIS & CO., London.—This addition to the various modes of applying adrenalin contains one part of that drug with fifty parts of chloretone in each 1,000 parts, and by thus combining the constringent, analgesic and antiseptic powers of its constituents forms a most effective remedy in all indolent inflammations, in hemorrhoids, pruritus ani, pruritus vulvæ, eczema, chronic congestions of the nose and ear, &c. In ophthalmic work it is useful in simple or purulent catarrhal inflammations. It is supplied in collapsible tubes with elongated tips, which facilitate nasal or rectal medication.

---

**Cholelith Pill (Pilula Cholelithica).**—PARKE, DAVIS & CO., London.—This chocolate-coated pill is a combination of oleate and silicylate of sodium with phenolphthalein and menthol. It is a new combination of drugs, which appears to have marked power to increase the amount of hepatic secretion, and to promote a due proportion of biliary acid salts wherewith to dissolve gall concretions, or at least so to diminish their size that they may be expelled naturally.

---

**Black Wash Tablets.**—PARKE, DAVIS & CO., London.—The provision of the chemical constituents of lotio hydrarg. nig. B.P.

in accurate proportions needing only to be treated as directed on the label, will be found a great convenience, particularly by medical officers to expeditions, to whom lime water may be a troublesome, if not unattainable, item.

---

**"Tabloid" Capsule Calcium Iodo-Ricinoleate, gr. 3 (0.194 gm.)**.—BURROUGHS, WELLCOME & CO., London.—Calcium iodo-ricinoleate is a new salt containing a large proportion of iodine, which combines the therapeutic value of calcium and of the iodides. It is tasteless and odourless, and is not affected by the gastric juice. It produces no digestive disturbance, and is well tolerated by patients who cannot take potassium or sodium iodide.

Calcium iodo-ricinoleate possesses in a marked degree the valuable action of its components, and in syphilitic cases the results obtained compare favourably with those of treatment by the iodides. It has proved very successful in condylomata and other specific manifestations, whilst in stubborn cases of ulcers—including rodent ulcer, which resisted other treatment—it has been reported to produce a healthy granulating surface. Various affections of lymphatic glands and of the thyroid have been favourably influenced by the administration.

One to three may be swallowed with water three or four times daily after food.

---

**"Phytin" Preparations.**—SOCIETY OF CHEMICAL INDUSTRY, Basle. London Agency, Harp Lane, E.C.—"Phytin" is stated to be the organic phosphorus-reserve material of green plants, and has been extracted from numerous seeds such as peas, haricot beans, and sunflower seeds.

It is a white powder, almost tasteless, and not very soluble in water.

Examination showed it to contain organically-combined phosphorus in large quantity.

It is supplied in capsules in the liquid form, and in combination with quinine.

For children it is prepared in combination with sugar of milk under the name of "Fortessan."

No doubt the various "Phytin" preparations will prove valuable adjuncts to the older phosphorus-containing organic compounds used in medicinal dietetics.

---

**Theinhardt's Food for Infants.**—THEINHARDT'S FOOD COMPANY, London, E.C.—This food has gained considerable popularity during recent years, and is no doubt one of the best on the market.

The makers claim it to be "The Ideal Food for Infants,"

this being partly based on a table giving the analyses of various proprietary foods.

We may point out that chemical analysis is not by any means the chief criterion of " food value," and many of the foods which are compared with " Theinhardt's " have proved themselves to be of great value in the diet of infants and invalids. We consider that any preparation should be judged on its own merits, and not on the doubtful demerits of others.

The analyses of various foods, compiled from Hutchison's and Konig's works on the subject, give very interesting results, but the table is too lengthy to reproduce. It shows that the Infantina food (Theinhardt's Soluble Infants' Food) contains a very high percentage of nitrogenous and fatty matters, and that the Hygiama is even richer in nutritive matter of the highest value.

We are informed that these foods are in constant use at the Milk Depots of Berlin and Charlottenburg. The Infantina is intended for infants from birth up to two years of age, and the Hygiama is for older children and adults.

They are excellent foods, worthy of all commendation. The Hygiama differs from the other food in that it is flavoured with cocoa, and may be taken either in solid form, or as a substitute for tea or coffee : when so used it forms a very palatable and nutritious beverage.

---

**" Manhu " Diabetic Foods.**—Manhu Food Company, Liverpool.—Under the above name several preparations are introduced for the use of diabetic subjects.

The firm claim that they have " changed " the starch without eliminating it, thus rendering it perfectly safe for diabetic patients.

This statement admits of criticism, for it is doubtful whether a physical or chemical change is referred to.

On several occasions we have chemically and microscopically examined several " Manhu " preparations, and found abundant evidence of the presence of starch, which gives the usual re-actions characteristic of this substance. Under these circumstances it is hard to understand the great superiority claimed for such preparations, as physicians can only condemn " diabetic " foods which actually contain starch grains in abundance.

We are of opinion that such foods as this for the diabetic are very misleading and dangerous. They are given to patients to whom starchy foods are forbidden, and the result is sometimes disastrous.

---

**" Miol."**—Miol Manufacturing Company, 66 Southwark Bridge Road, London, S.E.—Within the last few years numerous compounds, with malt extract as the chief ingredient, have been manufactured.

"Miol" appears to be one of the latest of these, a distinctive feature being that it contains, among other ingredients, the finest olive oil.

It is recommended as a valuable food in consumption and other diseases where cod liver oil is usually prescribed.

"Miol" is not unpleasant to take, the taste of the olive oil being partially covered by the malt; but we think it somewhat doubtful whether olive oil approaches cod liver oil for the cases in which the latter can be absorbed and assimilated.

"Miol" may prove of service in those cases where preparations of cod liver oil cannot be administered or digested.

